# Biomarkers in DILI: One More Step Forward

**DOI:** 10.3389/fphar.2016.00267

**Published:** 2016-08-22

**Authors:** Mercedes Robles-Díaz, Inmaculada Medina-Caliz, Camilla Stephens, Raúl J. Andrade, M. Isabel Lucena

**Affiliations:** Unidad de Gestión Clínica de Aparato Digestivo, Servicio de Farmacología Clínica, Instituto de Investigación Biomédica de Málaga, Hospital Universitario Virgen de la Victoria, Universidad de Málaga, Centro de Investigación Biomédica en Red de Enfermedades Hepáticas y DigestivasMálaga, Spain

**Keywords:** hepatotoxicity, drug-induced liver injury, prediction, diagnosis, outcome

## Abstract

Despite being relatively rare, drug-induced liver injury (DILI) is a serious condition, both for the individual patient due to the risk of acute liver failure, and for the drug development industry and regulatory agencies due to associations with drug development attritions, black box warnings, and postmarketing withdrawals. A major limitation in DILI diagnosis and prediction is the current lack of specific biomarkers. Despite refined usage of traditional liver biomarkers in DILI, reliable disease outcome predictions are still difficult to make. These limitations have driven the growing interest in developing new more sensitive and specific DILI biomarkers, which can improve early DILI prediction, diagnosis, and course of action. Several promising DILI biomarker candidates have been discovered to date, including mechanistic-based biomarker candidates such as glutamate dehydrogenase, high-mobility group box 1 protein and keratin-18, which can also provide information on the injury mechanism of different causative agents. Furthermore, microRNAs have received much attention lately as potential non-invasive DILI biomarker candidates, in particular miR-122. Advances in “omics” technologies offer a new approach for biomarker exploration studies. The ability to screen a large number of molecules (e.g., metabolites, proteins, or DNA) simultaneously enables the identification of ‘toxicity signatures,’ which may be used to enhance preclinical safety assessments and disease diagnostics. Omics-based studies can also provide information on the underlying mechanisms of distinct forms of DILI that may further facilitate the identification of early diagnostic biomarkers and safer implementation of personalized medicine. In this review, we summarize recent advances in the area of DILI biomarker studies.

## Introduction

Drug-induced liver injury (DILI) is one of many forms of adverse drug reactions that appear in a small proportion of patients. DILI is generally classified as intrinsic if predictable based on dose and pharmacological properties [for example acetaminophen (APAP) overdose] or idiosyncratic when unpredictable by the same features. The latter is believed to be a consequence of interactions between drug properties, host factors, and environmental conditions in a susceptible individual. Hence, no functional animal models are currently available for idiosyncratic DILI, unlike the presence of well-established murine models for APAP hepatotoxicity. DILI can have a profound impact on patient health due to its potential to cause acute liver failure, although most idiosyncratic DILI cases have a favorable outcome with full recovery after withdrawal of the culprit drug. DILI presents a clinical challenge as it can mimic almost any acute or chronic hepatobiliary condition and there are currently no specific biomarkers or diagnostic test available for this condition. A clinical diagnosis of idiosyncratic DILI therefore relies heavily on exclusion of alternative causes and a compatible drug history. The lack of DILI specific biomarkers also affects the drug development process and can result in early termination of drug candidates with assumed idiosyncratic hepatotoxicity potential. This can have considerable economic consequences and can prevent a large targeted recipient group from benefitting from a drug that may only be harmful to a small proportion of patients. New more specific and sensitive DILI biomarkers could enable better monitoring of patients receiving new drugs and minimize liver injury through early detection and subsequent cessation of dosing.

## Current Biomarkers in DILI

### Diagnosis

A biomarker is defined as a characteristic that is objectively measured and evaluated as an indicator of normal biological processes, pathogenic processes or pharmacologic responses to a therapeutic intervention (Biomarkers Definitions Working Group, 2001). While of uttermost importance in preclinical and early-phase clinical trial efficacy and safety evaluations of new drug compounds, biomarkers also play important roles in clinical practice to diagnose specific diseases, determine the state of a disease, disease prognosis and monitoring of clinical response to an intervention. Due to the lack of specific DILI biomarkers, DILI is monitored and diagnosed based on general liver injury serum biomarkers, such as alanine aminotransferase (ALT), aspartate aminotransferase (AST), alkaline phosphatase (ALP), and total bilirubin (TBL). ALT, AST, and ALP are all intracellular enzymes, which when detected in serum can indicate injury to hepatocytes or biliary cells. An increase in serum TBL on the other hand reflects liver dysfunction, but while being liver-specific it is relatively insensitive and subsequently appears later along the disease progression. ALT, AST, and ALP are not liver-specific and extrahepatic conditions such as myocardial damage, skeletal muscle diseases, hyperthyroidism, and bone diseases are associated with increased ALT, AST, or ALP. Interestingly, evidence of ALT and ALP plasma levels being influenced by genetic variations are also emerging (Yuan et al., 2008).

Alanine aminotransferase is thought to be more specific for liver injury than AST, as it is present mainly as a cytosolic protein in the liver and in low concentrations elsewhere, while AST is present in both cytosolic and mitochondrial forms in liver, heart, skeletal muscle, kidney, brain, pancreas, and lung tissue as well as in white and red blood cells (Giboney, 2005). Human ALT, however, exists as two isoforms with identical enzyme capacity, ALT1 and ALT2, although the presence of ALT2 appears to be restricted to non-hepatic tissue, in particular heart and skeletal muscles (Glinghammar et al., 2009). A recent study has demonstrated that assessing the percentage contribution of ALT1 and ALT2 activities to total ALT activity in plasma may distinguish hepatic from extrahepatic injury (Rafter et al., 2012). The standard ALT activity assay currently used in clinical practice, however, does not discriminate between serum ALT from different organs or isoforms

### Disease Phenotype

Drug-induced liver injury is generally classified into three patterns of liver injury (hepatocellular, cholestatic, and mixed), based originally on histological features. Hepatocellular type of injury is characterized by hepatocyte necrosis and inflammation, while bile stasis, portal inflammation and bile duct injury are commonly found in cholestatic injury. Mixed injury is, as the name suggests, a combination of these two liver injury types. As biopsies not normally form part of the DILI diagnostic process, the pattern of injury is mainly deduced based on liver profile analytical values. More precisely, the relationship between ALT and ALP values at DILI onset are used to determine the type of liver injury according to the R formula, R = (ALT/upper limit of normal, ULN) ÷ (ALP/ULN; Benichou, 1990; Aithal et al., 2011). An R value ≥ 5 is indicative of hepatocellular damage, R ≤ 2 cholestatic damage and 2 < R < 5 mixed damage. A recent comparison of ALT and AST elevations in a large DILI cohort found that AST can reliably substitute for ALT in R value calculations in cases were ALT values are absent at DILI onset, but that gamma-glutamyl transpeptidase (GGT) is less reliable as an ALP substitute (Robles-Diaz et al., 2015).

### Clinical Course and Prognosis

An ALT value alone offers little predictive value with regards to the clinical course of DILI, but can provide a prognostic indication when combined with TBL. This is today referred to as Hy’s Law and is based on clinical observations made by the late Hyman Zimmerman that DILI patients with hepatocellular type of liver injury and jaundice have a 10–50% higher risk of mortality or need for liver transplantation. Hy’s Law has since been defined more specifically as AST or ALT > 3 xULN and TBL > 2 xULN in the absence of ALP elevations and alternative reasons for ALT and TBL elevations, and is endorsed by the American Food and Drug Administration as criteria for severe DILI^1^.

The validity of Hy’s Law has now been confirmed in various large DILI cohorts (Andrade et al., 2005; Björnsson and Olsson, 2005; Chalasani et al., 2015). However, the effect of ALP increases on Hy’s Law have been questioned and recent findings demonstrate that an ALP level greater than 2 xULN does not have a protective role in acute liver failure development (Robles-Diaz et al., 2014). While having relatively high sensitivity, Hy’s Law lacks specificity as the majority of DILI cases meeting the Hy’s Law criteria in fact have a favorable outcome with full recovery. Using an extended range of analytical values we have developed a composite algorithm with higher specificity for the prediction of acute liver failure based on DILI onset data (Robles-Diaz et al., 2014).

## New Potential Biomarkers in DILI

The heightened interest in developing new more sensitive and specific DILI biomarkers for early prediction and diagnosis^2^^,^^3^^,^^4^ has resulted in the discovery of several promising candidates (**Table 1**). Many of these biomarker candidates were initially identified using APAP models, but could also be promising for aspects of idiosyncratic DILI.

**Table 1:** New serum/plasma DILI biomarker candidates.

Biomarker	Study cohort (N)	Comparison to alanine aminotransferase (ALT)	Reference
GLDH	Human: APAP overdose (33), cirrhosis and liver injury (108), hepatic carcinoma (40)	Overall correlation coefficient: 0.88. Lower correlation in APAP overdose than other liver injuries	Schomaker et al., 2013
GLDH	Human: APAP overdose (129)	Correlation coefficient (peak ALT): 0.45	Antoine et al., 2013
GLDH	Human: healthy volunteers receiving heparin	Correlation coefficient (peak ALT): 0.76	Harrill et al., 2012
MDH	Human: APAP overdose (33), cirrhosis and liver injury (108), hepatic carcinoma (40)	Overall correlation coefficient: 0.74. Less liver-specific than ALT.	Schomaker et al., 2013
HMGB1	Mouse: APAP overdose	Detectable elevations prior to ALT elevations. Peak value reached faster than ALT.	Antoine et al., 2009
HMGB1	Human: APAP overdose (84)	Correlation coefficient: 0.60. Acetylated HMGB1 associated with worse prognosis	Antoine et al., 2012
HMGB1	Human: APAP overdose (129)	Correlation coefficient (peak ALT): 0.67. Can predict clinical hepatotoxicity after APAP overdose prior to ALT.	Antoine et al., 2013
K18	Mouse: APAP overdose	Detectable elevations of FL-K18 and cK18 prior to ALT.	Antoine et al., 2009
K18	Human: APAP overdose (84)	Correlation coefficient: 0.58. Full length K18 associated with worse prognosis	Antoine et al., 2012
K18	Human: APAP overdose (129)	Correlation coefficient (peak ALT): FL-K18, 0.59; cK18, 0.57. FL-K18 can predict clinical hepatotoxicity after APAP overdose prior to ALT.	Antoine et al., 2013
K18	Humans: healthy volunteers receiving APAP (58), participating in an extreme adventure race (12)	Correlation coefficient (peak ALT): FL-K18, 0.70; cK18, 0.66. No elevation after muscular injury, which increased ALT	Thulin et al., 2014
miR-122, mir-192	Mouse: APAP overdose	Liver enriched miRs. Dose- and exposure duration-dependent changes in plasma detectable earlier than ALT.	Wang et al., 2014
miR-122, miR-192	Humans: APAP overdose (53)	Increased serum level after APAP overdose. miR-122 correlation coefficient (peak ALT): 0.46.	Starkey Lewis et al., 2011
miR-122	Humans: healthy volunteers receiving APAP (58), participating in an extreme adventure race (12)	Correlation coefficient (peak ALT): 0.62. No elevation after muscular injury, which increased ALT	Thulin et al., 2014
Eleven miRNAs profile	Humans: APAP overdose and ischemic hepatitis (49)	Diagnostic potential (elevated in APAP overdose but not in ischemic patients). Lack of miRNA profile recovery indicative of adverse patient outcome.	Ward et al., 2014

*GLDH, glutamate dehydrogenase; MDH, malate dehydrogenase; HMGB1, high-mobility group box-1 protein; K18, keratin-18; FL-K18, full length K18; cK18, caspase-cleaved fragment of K18; miR, microRNA.*

### Mechanistic-Based

Glutamate dehydrogenase (GLDH) is a mitochondrial enzyme found primarily in the liver and to a lesser degree in kidneys and trace amounts in skeletal muscles. This enzyme is more tissue-specific than ALT and AST. Healthy individuals present a stable and measurable level of serum GLDH that is unaffected by age and gender (Schomaker et al., 2013). A rise in circulating GLDH indicates mitochondrial dysfunction and subsequent loss of mitochondrial membrane integrity, which occurs during hepatocellular necrosis. GLDH correlates well with ALT elevations in patients with various forms of clinically demonstrated liver injury, including APAP overdose, but is not sensitive enough to predict APAP hepatotoxicity prior to ALT (Antoine et al., 2013; Schomaker et al., 2013). Furthermore, healthy volunteers receiving subcutaneous heparin injection have been reported to develop serum GLDH elevations in conjunction with asymptomatic ALT elevations, which underline the correlation between GLDH and ALT (Harrill et al., 2012). Similar results have been found for malate dehydrogenase (MDH), a constitutive enzyme in the citric acid cycle that is released into the serum after tissue damage (Schomaker et al., 2013). However, MDH is less tissue-specific and its serum level is subsequently affected by extrahepatic tissue damage.

High-mobility group box 1 (HMGB1) is a chromatin-binding protein with proinflammatory activity mediated through Toll-like receptor and receptor for advanced glycation endproducts signaling (Kubes and Mehal, 2012). It is passively released by necrotic cells in a hypoacetylated form, but not by apoptotic or secondary necrotic cells (Scaffidi et al., 2002). In contrast, it is actively secreted in a hyperacetylated form by immune cells (Bonaldi et al., 2003). Hence, HMGB1 is reflective of cell necrosis or activated immune cells depending on its acetylation status. Keratin-18 (K18) is a member of the keratin protein family, which is involved in cell structure and integrity. Similar to HMGB1, full-length K18 is released passively during cell necrosis. However, K18 undergoes caspase-mediated cleavage as part of structural rearrangements during apoptosis and can then be released into the blood stream (Ku et al., 2007). Hence, differentiating serum concentrations of full-length and caspase-cleaved K18 can provide an indication of the level of cell necrosis and apoptosis occurring in a subject. The molecular forms of HMGB1 and K18 have now been verified as sensitive blood-based markers of acute liver injury and provide insight into the mechanistic process of APAP hepatotoxicity (Antoine et al., 2009, 2012). These biomarkers have also been found to be more sensitive than ALT, with apparent elevations prior to ALT in patients at first presentation after APAP overdose intake (Antoine et al., 2013). The use of these biomarkers in clinical practice could potentially improve the speed of clinical decision-making. However, the value and clinical utility in cases with idiosyncratic DILI is still unknown.

### MicroRNA

MicroRNAs (miRNAs) are small (20–24 nucleotides) non-coding RNAs involved in post-transcriptional regulation of gene expression. Organ damage usually results in miRNAs being released into the bloodstream and to some degree also in urine. MiRNAs are relatively stable in biofluids, a feature that has contributed to that circulating miRNAs have received much attention lately as potential non-invasive DILI biomarker candidates. To date several studies have reported changes in serum miRNA concentrations during liver injury (Zhang et al., 2010; Starkey Lewis et al., 2011; Ward et al., 2014). MiR-122 and miR-192 were the first circulating miRNAs demonstrated to increase after toxic APAP doses in mice and soon after were confirmed to behave in a similar manner in human subjects (Wang et al., 2009; Starkey Lewis et al., 2011). MiR-122 and miR-192 are both liver-enriched miRNAs, with miR-122 variants accounting for approximately 72% of the total liver miRNA population in mice (Lagos-Quintana et al., 2002). Serum miR-122 is detectable at an early stage of hepatocellular damage as demonstrated in hospital admitted patients shortly after APAP overdose intake when ALT still remained normal (Antoine et al., 2013). This feature makes miR-122 particularly interesting for use during early phase human trials to detect drug candidates with hepatotoxicity potential, which may go undetected with the current biomarkers. Furthermore, miR-122 is more liver-specific than ALT as demonstrated in a human cohort with exercise-induced muscular injury, in which miR-122 remained stable while increases in ALT were detected (Thulin et al., 2014).

Emerging data also indicate that miRNA-122 could have a prognostic value, with higher early serum levels reported in APAP overdose patients who met the King’s College Criteria for liver transplantation (Starkey Lewis et al., 2011). However, the value of miR-122 for idiosyncratic DILI remains to be determined. It is interesting to note that while miR-122 shows much promise as a sensitive early blood borne DILI biomarker, urine levels of this miRNA does not increase notably after APAP overdoses neither in rats nor in humans (Yang et al., 2012, 2015). Recent data also suggest that serum miRNA profiles, rather than individual miRNAs, could have a higher diagnostic value. This is supported by a comparison of an 11-miRNA panel in acute liver injury patients of different etiologies (Ward et al., 2014).

### Exosomes

While miRNAs can enter the blood stream passively during cell necrotic and/or apoptotic cell death, active release also occurs in a regulated manner via exosomes. Exosomes are one of several forms of membrane-surrounded structures released by almost all types of cells. The complete role of exosomes besides transportation and delivery of various substances, such as signaling molecules and cellular waste, is yet to be elucidated (Yang et al., 2014). The constituents of exosomes are not limited to miRNAs but also include proteins, lipids, and additional nucleic acids (mRNAs). Exosomes have received much attention lately as a potential source for DILI biomarker explorations, seeing that exosome constituents change under cellular stress conditions (Wetmore et al., 2010; de Jong et al., 2012). Circulating exosomes originating from various tissues are found in many types of body fluids, with blood and urine being the more interesting sources for non-invasive biomarkers. A major challenge is subsequently to determine hepatocyte-derived exosomes. Nevertheless, promising results have emerged using animal models to demonstrate increases in exosomal liver-specific mRNAs (e.g., albumin, fibrinogen B β-polypeptide, haptoglobin, and β-actin) and liver-enriched miRNAs (miR-122 and miR-155) during acute liver injury (Wetmore et al., 2010; Bala et al., 2012). Interestingly, while increased levels of miR-122 and miR-155 were detected in circulating exosome-rich serum/plasma fractions in mice with alcohol- or lipopolysaccharide-induced liver injury, these miRNAs were found to be elevated in protein-rich serum/plasma fractions in mice with APAP hepatotoxicity, suggesting that miRNA compartment distribution pattern could also differ depending on the etiology (Bala et al., 2012). Variations in exosome-derived miRNA expression profiles have also been reported for additional liver conditions, such as chronic viral hepatitis B and C and non-alcoholic steatohepatitis compared to healthy controls. Furthermore, the miRNA expression pattern was found to reflect fibrosis stage and grade of liver inflammation in patients with chronic viral hepatitis C (Murakami et al., 2012).

### “Omics” Derived Biomarkers

The introduction of omics technologies offers a new approach for biomarker exploration studies. The ability to screen a large number of molecules (metabolites, proteins, DNA, etc.) simultaneously enables the identification of ‘toxicity signatures’, which could be used to enhance preclinical safety assessments and disease diagnostics. Omics-based studies can also provide information on the underlying mechanisms of distinct forms of DILI that could further facilitate the identification of early diagnostic biomarkers. Metabolomics is recognized as a promising technique, typically through mass spectrometry or nuclear magnetic resonance spectroscopy. This technique enables the evaluation of global metabolic changes and subsequently the identification of specific metabolic profiles associated with for example diseases or treatment responses. Metabolomics has been successfully applied to identify potential biomarker candidates in many types of liver diseases, including non-alcoholic fatty liver disease, steatosis, fibrosis, cirrhosis, hepatocellular carcinoma, and cholangiocarcinoma (reviewed by Beyoğlu and Idle, 2013). Furthermore, a comparison of plasma metabolic profiles of patients diagnosed with autoimmune hepatitis, primary biliary cirrhosis, autoimmune hepatitis/primary biliary cirrhosis overlap syndrome, DILI and healthy controls resulted in a metabolic autoimmune hepatitis phenotype with >93% sensitivity and specificity (Wang et al., 2014). Similarly, serum γ-glutamyl dipeptide levels have been demonstrated as potential biomarkers capable of discriminating between different forms of hepatic diseases (Soga et al., 2011).

However, in the field of hepatotoxicity, metabolomics studies performed to date are mainly limited to intrinsic DILI in animal or *in vitro* models. An ultra-performance liquid chromatography/time-of-flight tandem mass spectrometry analysis of galactosamine-treated rat sera found differences in several metabolites such as glucose, amino acids, and membrane lipids compared with control rats, with some of these correlating with the degree of histologically determined liver damage (Gonzalez et al., 2012). A similar study of sera from drug-treated rats found increases in bile acid levels in addition to several other metabolite differences, which combined with conventional clinical chemistry markers may improve the robustness and accuracy of a hepatotoxicity diagnosis (Buness et al., 2014). Recent animal studies also support that metabolic profiles, rather than individual metabolites, can predict hepatotoxicity, including idiosyncratic hepatotoxicity (Sun et al., 2014; Li et al., 2016). Metabolic profiling in urine has also been performed in search for metabolites associated with DILI. Healthy volunteers administered non-toxic levels of APAP presented both urine and plasma metabolic alterations when comparing pre- and post-dosing samples, which were more sensitive than changes in serum biochemical parameter over the study period (Kim et al., 2013). It should be pointed out that while many metabolic studies to date coincide in detecting variations in endogenous metabolites, the identified metabolites often vary between studies. Differences in study design as well as treatments could lead to such variations. It is also important to keep in mind that interindividual differences are common in metabolomics due to this method being very sensitive and subsequently can reflect variations in environmental and host conditions between subjects. Nevertheless, metabolomics could be a practical method for identifying DILI patients shortly after starting a new drug treatment and subsequently minimize serious liver injury through early treatment discontinuation when specific metabolic changes are detected. In addition to metabolomics, proteomics can be useful in the search for more specific DILI biomarkers. An exploratory comparison of global serum proteomes in DILI has reported promising results with apolipoprotein E expression demonstrating the greatest power to differentiate DILI from controls (Bell et al., 2012), while cadherin 5 and fatty acid binding protein 1 were found to be associated with DILI using an antibody bead array approach (Mikus et al., 2016). Likewise, genome-wide association studies of DILI cohorts have provided a number of specific HLA alleles that appear to be associated with distinct forms of DILI. These alleles, which have high negative predictive values, may be used as biomarkers to rule out DILI or identify the correct causative agent in patients taking more than one hepatotoxic agent, and subsequently facilitate the DILI diagnostic process (Aithal, 2015). However, the DILI forms associated with HLA risk alleles to date are limited to more common causative agents and many forms of DILI consequently lack determined HLA associations.

## Future Perspectives

Alanine aminotransferase, aspartate aminotransferase, alkaline phosphatase, and total bilirubin are currently the only approved DILI biomarkers in clinical practice. While fulfilling an important role in disease diagnostics, these biomarkers are not specific for hepatotoxicity, as an increase in any of these biochemical values is detected during practically all liver conditions. Furthermore, these biomarkers lack sensitivity as they appear once liver damage has developed and are consequently of limited use for predicting potential liver injury at a very early stage. This is of particular importance in clinical trials during drug development. The presence of DILI biomarkers with enhanced specificity and sensitivity may detect drug candidates with hepatotoxicity potential earlier in the drug development process and subsequently reduce the number of late stage drug attrition and postmarketing drug withdrawals. Furthermore, such biomarker would improve patient safety, due to offering improved monitoring during drug treatment initiation, as well as a faster and more accurate DILI diagnosis.

The lack of specific DILI biomarkers is to a large extent a consequence of the limited mechanistic understanding of this condition. The arrival of the “omics era” has brought hope and anticipation of scientific breakthroughs on the mechanistic side of DILI. Substantial progress has been made recently with the identification of new mechanistic-based biomarker candidates, such as HMGB1, K18, and miR-122. However, further efforts to explore and refine new improved DILI biomarker panels are needed. To be valid in clinical practice such non-invasive biomarkers not only need to demonstrate substantial specificity and sensitivity, but also need to be reasonably stable in extracted body fluids to allow reliable detection, as well as being easily assayed. The ability to validate new DILI biomarker candidates is restricted due to the current lack of fully functional animal models for idiosyncratic DILI. Although improved cell culture system such as induced pluripotent stem cell-derived hepatocytes are emerging, large DILI patient cohorts are the foremost approach for biomarker validations. This highlights the importance of collaborative efforts to establish DILI registries, which can circumvent the low DILI incidence rate generally encountered in individual hospital units. The Pro-Euro-DILI Registry is a recently established international DILI registry with serial biological sample collections from DILI onset to normalization of enrolled patients, intended for future DILI biomarker studies and validations (Slim et al., 2016).

## Author Contributions

All authors listed have made substantial, direct and intellectual contribution to the work, and approved it for publication.

## Conflict of Interest Statement

The authors declare that the research was conducted in the absence of any commercial or financial relationships that could be construed as a potential conflict of interest.

## Footnotes

1 http://www.fda.gov/downloads/Drugs/.../Guidances/UCM174090.pdf

2 http://www.imi-safe-t.eu/

3 http://www.mip-dili.eu

4 https://c-path.org/programs/pstc/
